# Patterns of domestic exposure to carbon monoxide and particulate matter in households using biomass fuel in Janakpur, Nepal^☆^

**DOI:** 10.1016/j.envpol.2016.08.074

**Published:** 2017-01

**Authors:** S.E. Bartington, I. Bakolis, D. Devakumar, O.P. Kurmi, J. Gulliver, G. Chaube, D.S. Manandhar, N.M. Saville, A. Costello, D. Osrin, A.L. Hansell, J.G. Ayres

**Affiliations:** a Institute of Applied Health Research, University of Birmingham, Birmingham, B15 2TT, UK; b UK Small Area Health Statistics Unit, MRC-PHE Centre for Environment and Health, Department of Epidemiology and Biostatistics, School of Public Health, Imperial College, London W2 1PG, UK; c UCL Institute for Global Health, University College London, 30 Guilford Street, London WC1N 1EH, UK; d Clinical Trial Service Unit and Epidemiological Studies Unit (CTSU), Nuffield Department of Population Health, Old Road Campus, Oxford OX3 7LF, UK; e Mother and Infant Research Activities (MIRA), Kathmandu 44600, Nepal; f Imperial College Healthcare NHS Trust, London, UK; g Institute of Occupational and Environmental Medicine, University of Birmingham, Edgbaston, Birmingham B15 2TT, UK; h Department of Biostatistics, Institute of Psychiatry, Psychology and Neuroscience, De Crespigny Park, London SE5 8AF, UK; i Department of Health Services and Population Research, Institute of Psychiatry, Psychology and Neuroscience, De Crespigny Park, London SE5 8AF, UK

**Keywords:** Household air pollution, Exposure assessment, Biomass, Carbon monoxide, Particulate matter, Nepal

## Abstract

Household Air Pollution (HAP) from biomass cooking fuels is a major cause of morbidity and mortality in low-income settings worldwide. In Nepal the use of open stoves with solid biomass fuels is the primary method of domestic cooking. To assess patterns of domestic air pollution we performed continuous measurement of carbon monoxide (CO) and particulate Matter (PM_2.5_) in 12 biomass fuel households in Janakpur, Nepal. We measured kitchen PM_2.5_ and CO concentrations at one-minute intervals for an approximately 48-h period using the TSI DustTrak II 8530/SidePak AM510 (TSI Inc, St. Paul MN, USA) or EL-USB-CO data logger (Lascar Electronics, Erie PA, USA) respectively. We also obtained information regarding fuel, stove and kitchen characteristics and cooking activity patterns. Household cooking was performed in two daily sessions (median total duration 4 h) with diurnal variability in pollutant concentrations reflecting morning and evening cooking sessions and peak concentrations associated with fire-lighting. We observed a strong linear relationship between PM_2.5_ measurements obtained by co-located photometric and gravimetric monitoring devices, providing local calibration factors of 4.9 (DustTrak) and 2.7 (SidePak). Overall 48-h average CO and PM_2.5_ concentrations were 5.4 (SD 4.3) ppm (12 households) and 417.6 (SD 686.4) μg/m^3^ (8 households), respectively, with higher average concentrations associated with cooking and heating activities. Overall average PM_2.5_ concentrations and peak 1-h CO concentrations exceeded WHO Indoor Air Quality Guidelines. Average hourly PM_2.5_ and CO concentrations were moderately correlated (r = 0.52), suggesting that CO has limited utility as a proxy measure for PM_2.5_ exposure assessment in this setting. Domestic indoor air quality levels associated with biomass fuel combustion in this region exceed WHO Indoor Air Quality standards and are in the hazardous range for human health.

## Highlights

• Intensive PM_2.5_ and CO monitoring was performed in 12 biomass fuel-using households in Janakpur.
• Domestic cooking activity patterns and kitchen, stove and fuel characteristics were recorded.
• Temporal PM_2.5_ and CO patterns demonstrated diurnal variability reflecting morning and evening cooking sessions.
• Average PM_2.5_ and peak CO concentrations both exceeded World Health Organization (WHO) Indoor Air Quality Guidelines.
• Concentrations of PM_2.5_ and CO were moderately correlated during cooking periods.

## 1 Introduction

Household Air Pollution (HAP) is a major global cause of morbidity and mortality, estimated to be responsible for 3.5 million premature deaths each year (Lim et al., 2012). The greatest disease burden is in low-income countries, due to reliance upon coal and biomass fuels including wood, crop residues and animal dung as the principal energy source for domestic cooking, heating and lighting (Bruce et al., 2000, Rehfuess et al., 2006). Solid fuel combustion results in emission of harmful airborne pollutants including particulate matter (PM), carbon monoxide (CO), and other toxic organic compounds (Fullerton et al., 2008, Smith, 2002). In Nepal it is estimated that approximately 80% of households use biomass as the principal source of domestic energy (Central Bureau of Statistics, 2011), with cooking typically performed on traditional open stoves with limited household ventilation. Studies in the Himalaya valley region and Dhanusha district have reported domestic PM levels which exceed World Health Organization (WHO) and National Ambient Air Quality Standards (Devakumar et al., 2014a) and the US EPA (Environmental Protection Agency) outdoor air quality index (Kurmi et al., 2008). However, there remains limited information regarding the characteristics and determinants of daily exposure patterns (Gurung and Bell, 2013).

Large-scale exposure assessment of household PM concentrations in low-income settings presents a number of methodological challenges. Gravimetric assessment methods necessitate resource intensive pre and post filter weighing (Northcross et al., 2015) and real-time measurements require bulky and expensive photometric devices. In contrast, CO can be measured using portable, real-time electrochemical devices (Smith et al., 2010). The utility and reliability of CO as a tracer for estimating PM exposure has been investigated in several low-income settings. Research conducted in open woodfire households in the Guatemalan highlands identified a strong correlation between CO and PM concentrations over long averaging times (Naeher et al., 2000, Naeher et al., 2001). Subsequent studies conducted in Guatemala (McCracken et al., 2013, Northcross et al., 2010) and Tibet (Li et al., 2012) also suggested a strong correlation in the context of a single dominant fuel source. However, concerns have been raised regarding wider applicability of CO as a surrogate measure for PM_2.5_ in large-scale epidemiological studies, due to influences of the indoor microenvironment (fuel, stove, cooking and kitchen characteristics) on the temporal and spatial relationship between pollutant concentrations (Clark et al., 2010, Dasgupta et al., 2009, Ezzati et al., 2000). Only a moderate correlation has been observed in studies conducted among biomass fuel households in Nepal, Peru and Kenya (Klasen et al., 2015), The Gambia (Dionisio et al., 2008) and Burkino Faso (Yamamoto et al., 2014, Pearce et al., 2009).

In this study, we sought to report patterns of domestic air pollution; to describe kitchen characteristics associated with indoor air quality and to characterise the relationship between kitchen CO and PM_2.5_ levels in a convenience sample of traditional biomass fuel households in southern Nepal.

## 2 Materials and methods

### 2.1 Setting

**Fig. 1:**
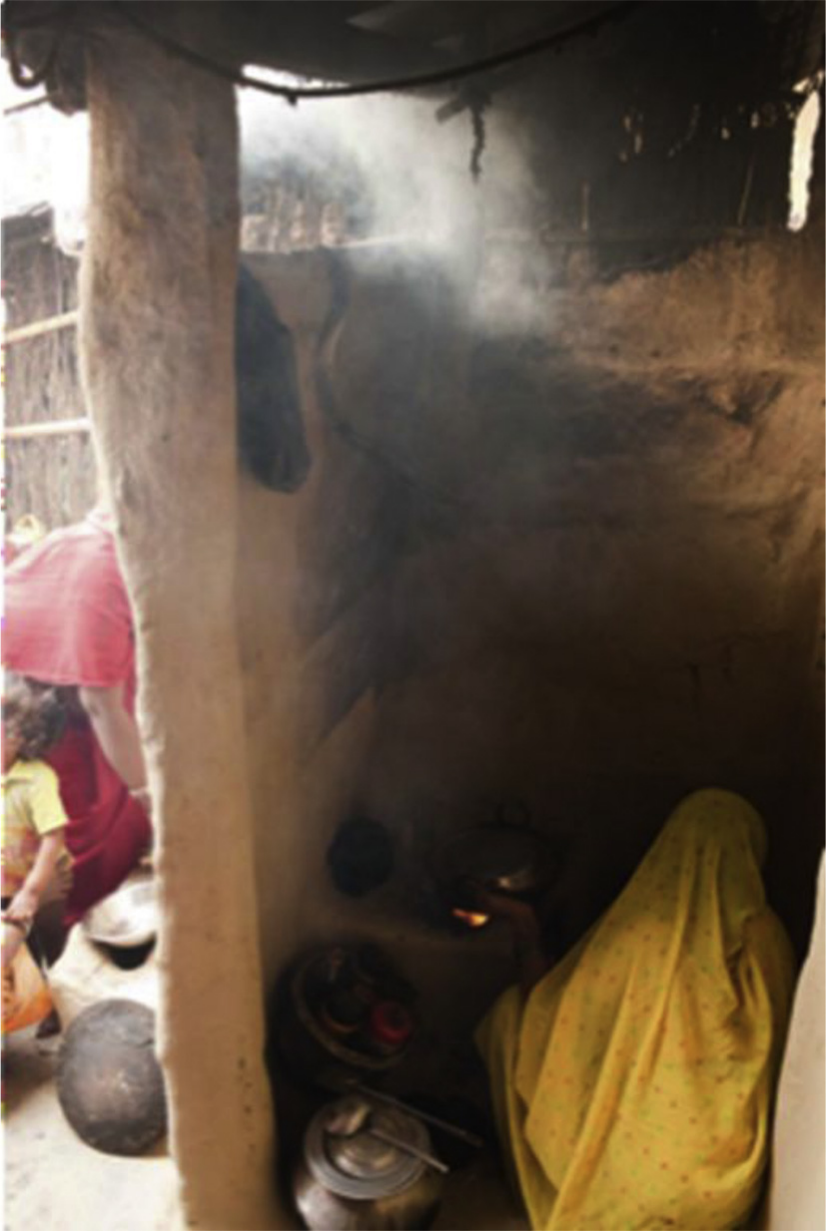
Typical study kitchen in Janakpur showing cooking stove (single pothole) located in the corner, open entrance door and eaves gap above the stove. The household member (mother) is performing cooking activities (evening meal preparation).

**Fig. 2:**
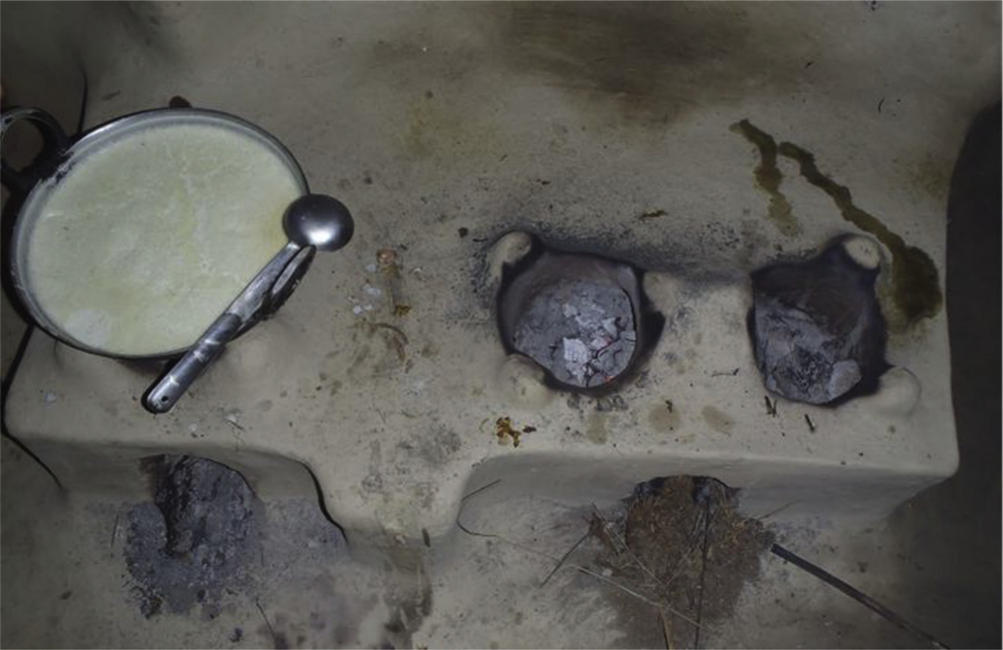
Typical biomass cooking stove (three pothole) at a morning cooking session.

This study was conducted in the Janakpur, the capital town of Dhanusha district in southern Nepal. Janakpur has a population of approximately 100,000 (Central Bureau of Statistics (2011)), comprising a central urban area with few asphalted roads and rural outskirts with minimal vehicular access. Fieldwork was conducted during the winter season, with temperature range 7 °C–23 °C and frequent heavy fog. In the study area, houses are typically constructed of dirt or brick on a timber frame, with an internal kitchen and open eaves space providing informal ventilation (Fig. 1). Traditional open stoves are widely used, consisting of a raised area with one to three potholes, above which a pan is placed (Fig. 2). The stove is usually lit for two sessions daily, to prepare late morning and early evening meals consisting of *dhal bhat* (rice and lentils), vegetables and warm buffalo milk. In the winter season the stove or fire in the central kitchen area may be lit overnight to provide warmth for kitchen livestock (goats, lambs, cattle).

### 2.2 Sampling strategy

The households in the study were a subset of homes of mothers recruited into a randomised controlled trial examining the effect of multiple micronutrient supplementation during pregnancy on child growth outcomes (Osrin et al., 2005, Devakumar et al., 2014b). At the second follow-up, a questionnaire was conducted to obtain household social, demographic and health information about participants and their families. Using this information, we identified a convenience sample of 12 households in which biomass fuels, including wood (mango/sal), dung (goitha) and mixed (wood/dung/straw) were exclusively used for household cooking and heating, no smokers were present in the household and an accessible electrical socket (220–240 V AC) was present in the kitchen.

### 2.3 Data collection

#### 2.3.1 Kitchen characteristics and cooking activity

The study fieldworkers observed and recorded information on kitchen construction (wall and roof type) and characteristics (windows, doors, eaves spaces) in addition to obtaining measurements of the stove (pan height) and kitchen floor, window and door areas. A ventilation index was calculated for each kitchen using a composite score for each open kitchen door (1) and window (0.5) (Dasgupta et al., 2009). Information on fuel selection and cooking patterns was obtained by questionnaire administered orally in Maithili (the local language), with direct translation of responses into English by trained fieldworkers. The cooking session was defined as the period from fire-lighting (start) to fire-extinguishing (end), obtained from self-report by household members with validation by direct observation of two complete cooking cycles in two households and twice-daily visits at all households.

#### 2.3.2 Carbon monoxide

Real-time CO levels were measured at 1-min intervals using an electrochemical EL-USB-CO monitor (Lascar Electronics Ltd, Erie, PA) with lower Limit of Detection (LOD) 3 ppm. Monitoring was performed in a central location in each study kitchen at height 50 cm and distance 150 cm from the cooking stove. Monitors were calibrated in the UK using >99.9%N_2_ (zero gas) and 100 ppm CO gas before transportation in airtight containers to Nepal with repeat calibration upon return to the UK. All monitoring commenced between 08:00 and 09:00 and was performed at one-minute intervals for a continuous period of approximately 48 h (range 45–50 h). All CO measurements below the LOD were assigned a value of one-half of the LOD (1.5 ppm). Data were downloaded using EasyLog USB software (Lascar Electronics Ltd).

#### 2.3.3 Particulate matter

Kitchen PM_2.5_ concentrations were estimated using the TSI DustTrak II 8530 (detection range 0.001–400 mg/m^3^) or AM510 SidePak (detection range 0.001–20 mg/m^3^aerosol monitoring device (TSI Inc, St. Paul MN, USA)). The internal laser photometers of these devices measure airborne particle mass concentration using a 90° light-scattering laser diode. The monitors were co-located (within 10 cm distance) and time-synchronised with the Lascar EL-USB-CO device to measure PM_2.5_ at 1-min intervals. Sampling was performed using an upstream external size-selective PM_2.5_ inlet attachment, with a level greased well as the impaction surface. The monitors were calibrated to a zero filter before and after each sampling period. Continuous PM_2.5_ monitoring was performed in eight households for approximately 48 h (range 42–48 h) and for a shorter duration in three households (range 22.5–32.5 h), with reliance upon rechargeable batteries due to scheduled power cuts (load shedding) precipitated by electricity supply shortages in Nepal. Data were downloaded from the photometric devices using TSI Trakpro software (TSI Inc, St Paul MN, USA).

### 2.4 Gravimetric calibration

Particulate matter concentration measured by light scattering devices is subject to measurement error since standard dust calibration is performed using artificial aerosols with different physical parameters (shape, size, density) to local cooking particles. To obtain a local ‘calibration factor’ as a reference to correct the photometric measurements, we performed simultaneous gravimetric sampling using the DustTrak filter facility (nine households) and using a co-located Casella Apex (Casella, Bedford, UK) gravimetric sampling device (four households). All gravimetric sampling was conducted in accordance with standard “Methods for Determination of Hazardous Substances (MDHS) no. 14/3 guidelines” (Health and Safety Executive, 2000). Samples were collected using new glass fiber 37 mm filters (Casella, Bedford, UK). All filters were weighed pre- and post-sampling on a Sartorius balance (Sartorius Ltd, Epsom, UK) accurate to 10 μg and calibrated at annual interval, in a temperature and humidity controlled environment maintained at the Department of Occupational and Environmental Medicine, University of Birmingham. Pre- and post-sampling weighing sessions were performed over two consecutive days, following a 24-h period of acclimatisation at room temperature. Study filters were maintained in a protective plastic sleeve (SKC Ltd., Dorset UK) and stored inside an airtight container for transportation to Nepal. Five filters were used as field blanks to correct for changes in filter weight due to exposure to study conditions.

**Fig. A.1:**
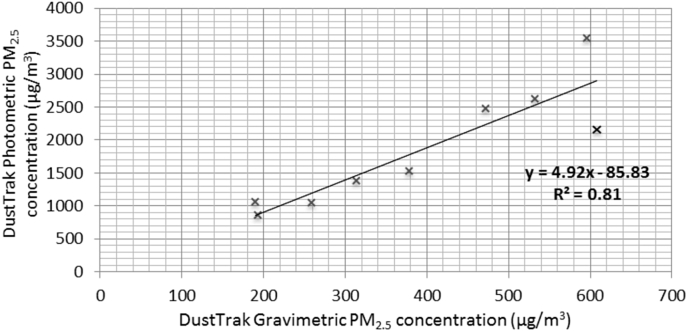
Relationship between PM_2.5_ (μg/m^−3^) measured by gravimetric and photometric sampling (DustTrak device).

**Fig. A.2:**
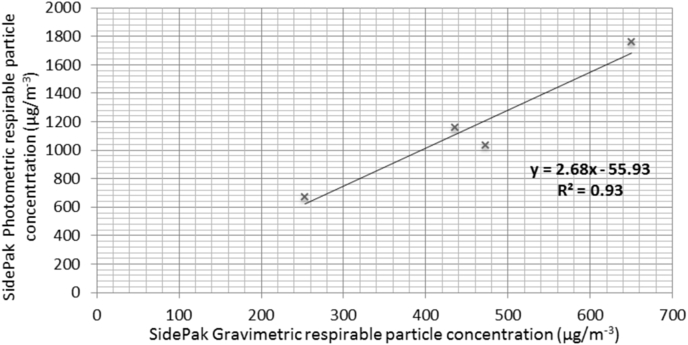
Relationship between PM_2.5_ (μg/m^−3^) measured by gravimetric and photometric sampling (SidePak device).

Air sampling was performed using an Apex air pump attached to a PM_2.5_ cyclone head (Casella, Bedford UK) using a portable flow meter with a flow rate 2.2 L/min (Casella Ltd, Rotameter, range 0.5–5 L/min). Flow rate calibration was performed with a Bios Dry Cal DC-Lite Primary flowmeter in the UK. The Apex monitor was co-located within 10 cm of the photometric device, with simultaneous gravimetric monitoring performed for up to 48 h (mean duration 20 h). We calculated a local calibration factor using the ratio of average PM_2.5_ concentrations obtained by each method (Dionisio et al., 2008, Li et al., 2012), achieving a total of 13 paired measurements. There was a strong linear relationship between the photometric and gravimetric techniques; R^2^ = 0.81 (n = 8), providing a calibration factor of 4.9 (Appendix A: Fig. A.1); and similarly for the SidePak device (R^2^ = 0.93, n = 4), providing a calibration factor of 2.7 (Appendix A: Fig. A.2). Respective custom calibration factors were applied to all photometric PM_2.5_ observations prior to statistical analyses.

### 2.5 Statistical analysis

We calculated descriptive statistics for kitchen characteristics, cooking activity patterns and average (mean, median) CO and PM_2.5_ concentrations in study households, with real-time exposures averaged over the total sampling duration and cooking period duration respectively in each household. ANOVA was conducted to examine the difference in the mean values of CO and PM_2.5_ by predictive factors: fuel type (wood, dung mixed), wall material (dirt/brick/mixed), kitchen size (area <15.3 m^2^, area ≥15.3 m^2^), ventilation index (high/low), eaves space (<27 cm, ≥27 cm) and stove type (single/multiple). We conducted multiple linear regression to determine a set of variables to best explain variability in log-transformed air quality measurements during cooking sessions, with likelihood ratio tests used to determine final multivariate models. Pearson's correlation coefficients were calculated evaluate the correlation between 1-h average PM_2.5_ and CO levels. All statistical analyses were performed in Stata (Version 13; Stata Corp, USA, 2013).

### 2.6 Ethical approval

Ethical approval was granted by the Nepal Health Research Council and the UCL Research Ethics Committee. Written consent was obtained from participating mothers at the first point of contact and consent was obtained verbally at each sampling visit. Each participating family was reimbursed with 100 Nepalese Rupees and a packet of biscuits.

## 3 Results

### 3.1 Household characteristics and cooking activity

Detailed characteristics of the study kitchens are shown in Table 1. Cooking was performed indoors in all except one household (W2), in which the stove was located in a courtyard. Kitchens were constructed of mud/dirt on wooden frame, bricks or a mixed composition with median floor area 15.3 m^2^ (range 2.1–46.9 m^2^). There were no formal ventilation sources, but an open eaves gap provided informal ventilation in nine kitchens (median 27 cm, range 10–90 cm). Cooking stoves were all located against an external wall and consisted of single or multiple potholes. Meal preparation was performed twice daily by female family members, with children (age range 16 months to 8 years) present in the kitchen during cooking sessions in ten households. Morning cooking commenced between 07:00 and 08:00 with the stove lit for median duration 2.5 h (range 2–4 h), and a shorter evening session from 17:00 to 20:00 (median duration 1.5 h, range 1–2 h). An overnight fire was lit in five study households, to provide warmth for resident livestock.

### 3.2 Daily variations of PM_2.5_ and CO

**Fig. 3:**
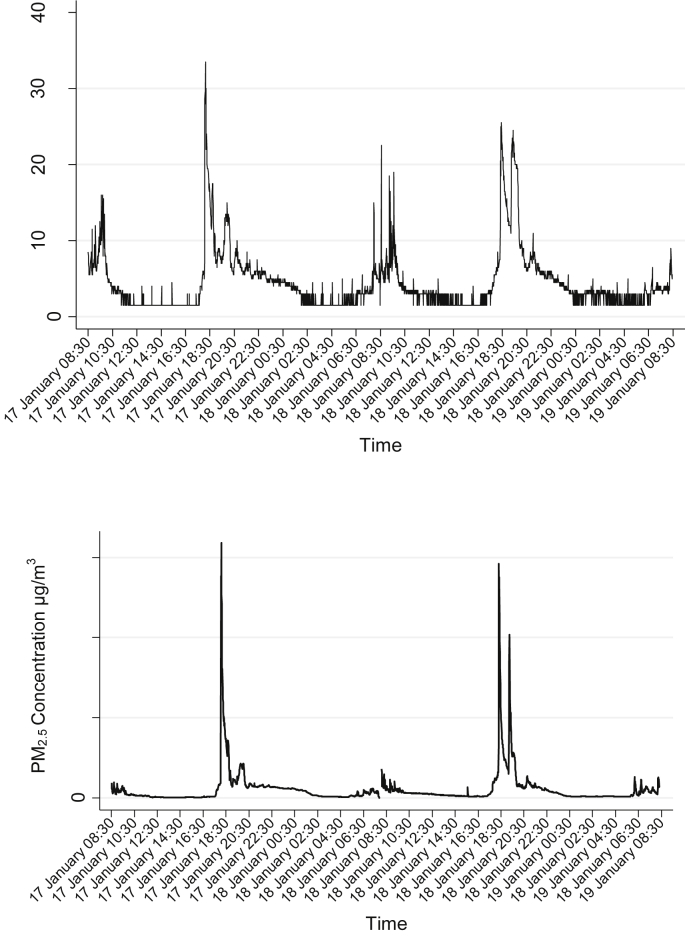
Diurnal variation of CO and PM_2.5_ (17–19 January 2012) in a typical study household (dung fuel).

Descriptive statistics for pollutant measurements by household are presented in Table 2, comprising a total of 34,400 and 27,450 CO and PM_2.5_ minute-level measurements respectively. Measured ranges of 48-h mean CO and PM_2.5_ levels by household were 3.0–6.8 ppm and 187.9–619.3 μg/m^3^, respectively. The continuous CO and PM_2.5_ concentration profile consistently showed diurnal peaks reflecting morning and evening cooking periods (Fig. 3). Extremely high peak 1-min average CO and PM_2.5_ concentrations typically coincided with lighting the stove for evening cooking, with maximum observed values of 162.5 ppm (wood fuel) and 15,900 μg/m^3^ (dung fuel), adjusted for calibration factor for CO and PM_2.5_ respectively.

### 3.3 Daily, cooking period and peak 1-h pollutant concentrations

Summary statistics developed by averaging duration and stove activity are presented in Table 3. Overall mean (SD) 48-h concentrations of CO and PM_2.5_ were 5.4 (4.3) ppm and 417.6 (686.4) μg/m^3^, respectively. Cooking activities were associated with elevated CO and PM_2.5_ concentrations of 8.3 (5.2) ppm and 966 (1384) μg/m^3^, respectively. Lighting of a kitchen fire to provide warmth for resident livestock was also associated with increased levels: mean CO concentration 6.3 (3.1) ppm and PM_2.5_ 527.4 (409) μg/m^3^. Peak 1-h CO concentrations were in the range 8.1–31.9 ppm, with average peak 1-h concentration exceeding the 48-h mean by an average factor of 3.1. Corresponding values for peak 1-h PM_2.5_ concentrations were in the range 566.4–3805.0 μg/m^3^, exceeding the 48-h mean value by an average factor of 4.2.

### 3.4 Fuel, kitchen and stove characteristics

Households using dung and mixed fuels had the highest average concentrations of CO and PM_2.5_ during cooking sessions, with the greatest variability in concentration magnitude associated with dung fuel. In ANOVA analyses, all kitchen and stove characteristics were significantly associated with average CO and PM_2.5_ concentrations during cooking periods (Table 4). Multivariable linear regression models using explanatory variables to predict log-transformed CO and PM_2.5_ concentrations were compared using likelihood ratio tests (Table 5, Table 6). The multivariable model including fuel type, wall material, ventilation index and kitchen area explained 17% of variation in natural logarithm transformed CO levels during cooking periods and 18% of variation in logarithm transformed PM_2.5_ concentration.

### 3.5 Correlation between average hourly kitchen PM_2.5_ and CO concentrations

**Fig. 4:**
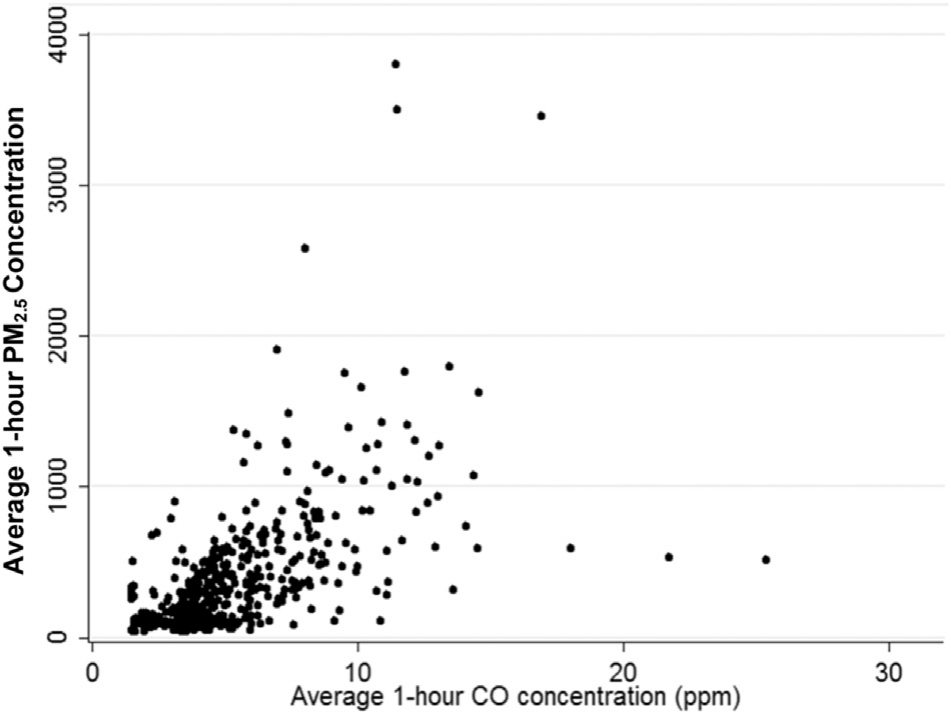
Scatterplot of hourly concentrations for PM_2.5_ and CO (r = 0.59).

Hourly average CO and PM_2.5_ concentrations were moderately correlated (*r* = 0.59; p < 0.001) (Fig. 4), more strongly for dung (r = 0.68; p < 0.001) and mixed (*r* = 0.62; p < 0.001), than for wood fuel households (*r* = 0.52, p < 0.001).

## 4 Discussion

Our real-time kitchen CO and PM measurements from 12 biomass fuel households in Janakpur, Nepal, provide evidence of domestic air pollution levels in this region that are in the hazardous range for human health (Smith, 2002). We report an average PM_2.5_ concentration of 418 μg/m^3^, which greatly exceeds the WHO Air Quality Guidance (AQG) Interim-Target-1 recommendation of 35 μg/m^3^ for household fuel combustion (WHO, 2014). Households using dung and mixed fuel sources had the highest peak concentrations and greatest variability of CO and PM_2.5._ The highest recorded peak 1-h CO concentration (31.9 ppm) exceeded the WHO AQG 60-min exposure guideline (30 ppm) (WHO, 2010), with maximum values (25.4 and 27.3 ppm) approaching the guideline limit in two additional households.

The observed temporal variation in pollutant patterns is consistent with findings from comparable settings with similar kitchen and stove characteristics (Ezzati et al., 2000, Naeher et al., 2001). Overall diurnal pollutant patterns were similar in pattern but lower in magnitude than those reported from the Sarlahi District of Nepal, where average 24-h pollutant concentrations of CO and PM _2.5_ of 9.1 ppm and 650 μg/m^3^ were measured respectively (Klasen et al., 2015) suggesting possible differences in local cultural cooking practices. Measured differences in pollutant concentrations between cooking and non-cooking sessions were consistent with those obtained for average PM_4_ concentrations in an associated study in Janakpur (Devakumar et al., 2014a) and for PM_2.5_ average concentrations reported in other low-income settings (Clark et al., 2010, Commodore et al., 2013).

To the best of our knowledge this is the first study to consider the relationship between CO and PM_2.5_ in biomass fuel households in the Dhanusha region of Nepal. We identified a moderate overall correlation between CO and PM_2.5_ concentrations during cooking sessions (r = 0.59), which is lower than the correlation coefficient of 0.92 between CO and PM_2.5_ concentrations in wood fuel households in Guatemala, yak dung stoves in Tibet (R^2^ = 0.87, r^2^ = 0.88) (Li et al., 2012) and by Dionisio and colleagues in The Gambia (r = 0.80) (Dionisio et al., 2008). Investigators in Burkino Faso reported only a weak correlation (Spearman ρ = 0.22) between PM_10_ and CO (Yamamoto et al., 2014). Such variation may be explained by the local cooking characteristics, including fuel type and cooking style or influences of the local microenvironment. Investigators have observed greater discordance at low pollutant concentrations (Klasen et al., 2015) and high PM variability for a single CO concentration (Pollard et al., 2014) suggesting a complex relationship between the two pollutants that is determined by a range of local factors. It has also been observed that the PM-CO relationship may be determined by housing characteristics and stove conditions that differentially influence the emission and dispersal of particle and gaseous pollutants (Naeher et al., 2001). Although CO has been applied as a surrogate measure (Northcross et al., 2015) our findings suggest limited utility as a proxy measure concentration in this setting. Furthermore, individual pollutant measurements are more informative for assessing different health risks, with PM_2.5_ widely associated with respiratory conditions and increasing evidence regarding an association between high CO exposure and adverse cardiovascular, neurodevelopmental and fetal outcomes (Dix-Cooper et al., 2012, Mustafic et al., 2012, Pope et al., 2010).

We observed higher average pollutant concentrations associated with mixed wall composition and a low ventilation index, suggesting a role for micro-environmental factors on overall average kitchen concentrations. An unexpected finding was that households with larger kitchens appeared to have higher mean PM_2.5_ and CO concentrations than those with a smaller floor surface area. These differences may be explained the location of stove which was frequently observed to be in a corner area close to the eaves space in small kitchens. We observed influences of stove activity patterns on CO and PM_2.5_, with peak pollutant concentrations associated with stove lighting, and return of concentrations to background values between cooking sessions. Previous studies have indicated that from a public health perspective increasing kitchen ventilation may be a low cost intervention to reduce HAP (Krieger and Higgins, 2002).

A key strength of our study was the availability of real-time monitoring at one-minute intervals over a 48-h period, providing over 60,000 individual measurements and enabling reporting of detailed temporal pollutant patterns including characterisation of peak exposure periods over four cooking cycles. We have also reported specific local calibration factors for the DustTrak and SidePak devices, which may be utilised for future photometric exposure assessment studies in this setting and detailed information regarding local cooking practices and kitchen characteristics.

The main limitation of the study was the small sample size, reflecting the practical and logistical challenges in conducting continuous pollutant monitoring in a low-income setting. It was not possible to achieve continuous monitoring for a full 48-h period in all study households due to restrictions on the timing of household visits and lack of access to a continuous electricity supply. The households were selected to represent a range of fuel types and to reflect traditional Nepalese cooking practices, improving applicability of our findings to similar domestic settings. Our study measurements were performed only in the winter season, limiting generalisability of our findings to the wet season and warmer summer months when different cooking activity patterns and kitchen ventilation practices may be observed. There are some limitations to our exposure assessment methodology: nocturnal CO concentrations were frequently low and results < LOD may be of limited utility (Helsel, 2010). Occasional PM_2.5_ concentrations exceeded the photometer upper detection limit, which may lead to under-estimation of mean concentration. We did however perform standardised data collection in each study household.

We did not attempt to measure personal exposure levels, but the monitoring location in all households reflected the position of the cook tending the fire and the site of highest peak household exposure for women and children (Commodore et al., 2013, Pearce et al., 2009). Exposure from lighting sources was unlikely to contribute significantly to pollutant levels as lights were generally supplied by electricity and limited to bedroom areas. We did not identify outdoor sources of PM and CO such as traffic and industrial emissions, but all study households were located away from major roads and there are few motorised vehicles or industrial sources of outdoor air pollution in Janakpur.

## 5 Conclusions

Our findings indicate that domestic CO and PM_2.5_ levels in biomass fuel households in this area of Nepal frequently exceed WHO Air Quality Standards and are likely to contribute to increased morbidity, mortality and adverse birth outcomes. Our results suggest that CO has limited utility as a proxy measure for accurate PM_2.5_ exposure assessment in similar traditional domestic settings.

## Role of the funding source

The study was made possible by a Wellcome Trust Research Elective Prize for SB and a Wellcome Trust Training Fellowship for DD (Grant number: 092121/Z/10/Z). The work of the UK Small Area Health Statistics Unit is funded by Public Health England as part of the MRC-PHE Centre for Environment and Health, funded also by the UK Medical Research Council (Grant number: MR/L01341X/1). The funders played no role in the conception, methodology, analysis or reporting of this study.

## Conflict of interest statement

The authors declare that they have no conflicts of interest.

## Figures and Tables

**Table 1:** Household sampling schedule and fuel, kitchen and stove characteristics.

Household^a^	Wall material	Kitchen area (m^2^)	Kitchen eaves (cm)	Kitchen ventilation index	Stove type	Sampling date (2012)
W1	Dirt	2.1	90	2	Single	14–16 Jan
W2^b^	Dirt	26.5		0	Single	15–17 Feb
W3	Dirt	2.6	27	1.5	Single	28–30 Jan
W4	Brick	5.7		1.5	Single	5–7 Feb

D1	Brick	15.1	14	1	Multiple	8–10 Feb
D2	Mixed	29.7	10	1	Multiple	20–22 Jan
D3^b^	Dirt	18.0	25	1	Multiple	17–19 Jan
D4^b^	Mixed	7.1	83	3.5	Multiple	31 Jan–2 Feb

M1^b^	Dirt	46.9	60	4	Multiple	24–26 Jan
M2	Dirt	15.5		2	Multiple	10–12 Feb
M3	Brick	7.0	68	1.5	Multiple	26–28 Jan
M4^b^	Dirt	16.6	20	0	Multiple	2–4 Feb

a W = wood fuel, D = dung fuel, M = mixed fuel.

b Night-time kitchen fire.

**Table 2:** Air quality summary statistics (TWA) by household.

CO (ppm)						PM_2.5_ (μg/m^3^)				
Household	Duration (hours)	Mean^b^ (SD)	Median (IQR)	Range	1-h maximum^c^	Duration (hours)	Mean (SD)	Median (IQR)	Range	1-h maximum^b^
W1	48	4.8 (1.4)	4.5 (3.5–6.0)	1.5–13.0	8.1	22.5	242.0 (204.2)	192.2 (91.8–340.0)	6.4–2880.0	566.4
W2^a^	47	6.8 (8.6)	5.0 (3.5–7.0)	1.5–162.5	31.9	–	–	–	–	–
W3	45	5.9 (5.1)	4.5 (3.5–6.5)	1.5–88.0	25.4	42	300.8 (315.0)	236.0 (88.2–410.7)	24.0–3829.5	1042.0
W4	48	4.7 (2.1)	4.0 (3.5–5.0)	1.5–20.0	11.1	45	187.9 (259.4)	145.0 (75.1–193.5)	40.7–3967.9	1097.7
D1	48	6.0 (5.1)	4.5 (3.5–6.0)	1.5–47.5	27.3	46	260.5 (598.4)	103.2 (75.1–189.8)	24.4–6775.4	1620.2
D2	50	5.8 (3.1)	4.5 (3.5–7.5)	1.5–22.0	11.8	32.5	303.0 (407.6)	187.2 (91.6–322.0)	42.0–5280.0	1044.1
D3^a^	48.5	4.5 (4.0)	3.5 (1.5–5.5)	1.5–33.5	16.9	48	543.2 (1165.3)	236.0 (99.4–592.0)	0–15900.0	3499.4
D4^a^	48	6.5 (4.2)	5.0 (3.5–8.0)	1.5–30.5	18.0	48	440.2 (541.5)	296.0 (150.0–469.0)	0–5564.4	1798.1
M1^a^	48	6.5 (2.6)	5.5 (4.5–8.0)	1.5–26.0	11.9	48	619.3 (808.8)	431.1 (276.4–692.6)	52.0–13,400.0	3805.0
M2	46.5	5.5 (2.8)	5.0 (4.0–6.5)	1.5–32.0	13.1	46	511.7 (711.4)	364.5 (156.9–536.5)	61.8–7383.4	1911.2
M3	49	3.0 (3.3)	1.5 (1.5–3.0)	1.5–55.5	12.2	32	500.2 (461.5)	310.0 (270.0–603.1)	130.6–5080.0	1760.2
M4^a^	48	4.7 (2.1)	4.0 (3.5–5.0)	1.5–20.0	11.1	47.5	399.1 (369.6)	317.5 (214.6–488.8)	127.7–6696.3	1281.7

SD: Standard deviation. IQR: interquartile range.

a Night-time kitchen fire.

b Arithmetic mean: time-weighted average (TWA) for the respective total sampling duration.

c Arithmetic mean: maximum 1-h value.

**Table 3:** Air quality summary statistics by averaging duration and stove activity.

Air quality measure	N	CO (ppm)			N	PM_2.5_ (μg/m^3^)		
		Mean (SD)	Median	Range		Mean (SD)	Median	Range
48-h average	12	5.4 (4.3)	4.5	1.5–162.5	8	417.6 (686.4)	260.5	0–15900.0
1-h maximum	12	16.5 (7.6)	12.6	8.1–31.9	11	1766.0 (1018.4)	1620.2	566.4–3805.0
**Stove Activity**								
Morning cooking	12	8.4 (5.4)	7.5	1.5–108.5	8	872.9 (1137.6)	539.5	0–13400.0
Evening cooking	12	8.0 (4.9)	7.0	1.5–162.5	8	1117.9 (1702.0)	616.0	0–15900.0
Heating^a^	5	6.3 (3.1)	5.5	1.5–36.0	4	527.4 (409.0)	456.0	52.0–8100.0
Non-cooking	12	4.8 (3.8)	4.0	1.5–30.0	8	329.1 (426.1)	226.0	0–10180.0

CO: carbon monoxide. PM_2.5_: Particulate Matter with diameter ≤2.5 μm. SD, Standard deviation.

a Night-time kitchen fire.

**Table 4:** Average (mean) cooking period CO and PM_2.5_ concentrations by fuel, kitchen and stove characteristics.

Kitchen and stove characteristics	N (households)	CO (ppm)		PM_2.5_ (μg/m^3^)	
Fuel type			p < 0.001		p < 0.001
Wood	4	7.6 (5.5)		520.0 (526.8)	
Dung	4	10.3 (5.3)		1179.4 (1766.5)	
Mixed	4	7.1 (4.4)		1037.2 (1442.3)	
Wall material			p < 0.001		p < 0.001
Dirt	7	8.4 (5.1)		1037.2 (1442.3)	
Brick	3	7.8 (6.2)		622.1 (995.1)	
Mixed	2	8.9 (2.7)			
Area			p < 0.001		p < 0.001
Large (>15.3 m^2^)	6	8.7 (5.0)		1132.8 (1646.5)	
Small (≤15.3 m^2^)	6	8.0 (5.4)		741.2 (872.0)	
Eaves			p < 0.001		p < 0.001
Wide (>27 cm)	4	7.7 (5.4)		1121.4 (1303.8)	
Narrow (≤27 cm)	5	9.0 (4.8)		852.3 (1430.8)	
Ventilation Index			p < 0.001		p < 0.001
High (≥1.5)	7	7.5 (4.4)		957.5 (1616.2)	
Low (≤1.5)	5	9.4 (5.9)		972.6 (1175.2)	
Stove type			p < 0.001		p < 0.001
Single	4	7.6 (5.4)		520.0 (506.8)	
Multiple	8	8.6 (5.1)		1073.2 (1502.6)	

ANOVA analysis: significance level = 0.05.

**Table 5:** Likelihood ratio selection for linear regression model: natural logarithm transformed CO concentrations during cooking sessions.

Model number	Variables in model	Model comparison (likelihood ratio)	p-value
1	Fuel type	–	–
2	Fuel type	Model 2 vs Model 1	p < 0.001
	Wall material		
3	Fuel type	Model 3 vs Model 2	p < 0.001
	Wall material		
	Ventilation index		
4	Fuel type	Model 4 vs Model 3	p < 0.001
	Wall material		
	Ventilation index		
	Kitchen area		

**Table 6:** Likelihood ratio selection for linear regression model: natural logarithm transformed PM_2.5_ concentrations during cooking sessions.

Model number	Variables in model	Model comparison (likelihood ratio)	p-value
1	Fuel type	–	–
2	Fuel type	Model 2 vs Model 1	p < 0.001
	Ventilation index		
3	Fuel type	Model 3 vs Model 2	p < 0.001
	Ventilation index		
	Wall material		
4	Fuel type	Model 4 vs Model 3	p = 0.002
	Ventilation index		
	Wall material		
	Kitchen area		

## Appendix A

### Gravimetric calibration

For gravimetric sampling the average filter pre-weight was subtracted from the average post-weight, with adjustment of the change in mass of the field blanks (total average weight change + 0.070 mg). Sample volume was calculated from the sampling duration and flow rate. Particulate concentration was calculated using Equation 1:

Equation 1: Concentration = (Average post-weight − Average pre-weight ± Δ field blank)/Volume

Real-time exposures were averaged over the corresponding photometric sampling duration to obtain a time weighted average (TWA) concentration. Co-located measurements were performed in eight households (DustTrak) and four households (SidePak). The paired data were used to determine custom calibration factors which indicated that the DustTrak overestimated gravimetric mass concentration by a factor of 4.9 (R^2^ = 0.81, *p* < 0.01, Fig. A.1) and the SidePak by a factor of 2.7 (R^2^ = 0.93). The calibration factors were applied to adjust all PM_2.5_ measurements recorded by photometric monitoring in the study.

## References

[bib1] Bruce N.R., Perez-Padilla R., Albalak R. (2000). Indoor air pollution in developing countries: a major environmental and public health challenge. Bull. World Health Organ..

[bib2] Central Bureau of Statistics (2011). National Population and Housing Census. http://unstats.un.org/unsd/demographic/sources/census/wphc/Nepal/Nepal-Census-2011-Vol1.pdf.

[bib3] Clark M.L., Reynolds S.J., Burch J.B., Conway S., Bachand A.M., Peel J.L. (2010). Indoor air pollution, cookstove quality, and housing characteristics in two Honduran communities. Environ. Res..

[bib4] Commodore A.A., Hartinger S.M., Lanata C.F., Mäusezahl D., Gil A.I., Hall D.B., Aguilar-Villalobos M., Naeher L.P. (2013). A pilot study characterizing real time exposures to particulate matter and carbon monoxide from cookstove related woodsmoke in rural Peru. Atmos. Environ..

[bib5] Dasgupta S., Wheeler D., Hug M., Khaliquzzaman M. (2009). Improving indoor air quality for poor families: a controlled experiment in Bangladesh. Indoor Air.

[bib7] Devakumar D., Semple S., Osrin D., Yadav S.K., Kurmi O.P., Saville N.M., Shrestha B., Manandhar D.S., Costello A., Ayres J.G. (2014). Biomass fuel use and the exposure of children to particulate air pollution in southern Nepal. Environ. Int..

[bib6] Devakumar D., Chaube S.S., Wells J.C., Saville N.M., Ayres J.G., Manandhar D.S., Costello A., Osrin D. (2014). Effect of antenatal multiple micronutrient supplementation on anthropometry and blood pressure in mid-childhood in Nepal: follow-up of a double-blind randomised controlled trial. Lancet Glob. Health.

[bib8] Dionisio K.L., Howie S., Fornace K.M., Chimah O., Adegbola R.A., Ezzati M. (2008). Measuring the exposure of infants and children to indoor air pollution from biomass fuels in the Gambia. Indoor Air.

[bib9] Dix-Cooper L., Eskenazi B., Romero C., Balmes J., Smith K.R. (2012). Neurodevelopmental performance among school age children in rural Guatemala is associated with prenatal and postnatal exposure to carbon monoxide, a marker for exposure to woodsmoke. Neurotoxicology.

[bib10] Ezzati M., Saleh H., Kammen D.M. (2000). The contributions of emissions and spatial microenvironments to exposure to indoor air pollution from biomass combustion in Kenya. Environ. Health Perspect..

[bib11] Fullerton D.G., Bruce N., Gordon S.B. (2008). Indoor air pollution from biomass fuel smoke is a major health concern in the developing world. Trans. R. Soc. Trop. Med. Hyg..

[bib12] Gurung A., Bell M.L. (2013). The state of scientific evidence on air pollution and human health in Nepal. Environ. Res..

[bib13] Health and Safety Executive (2000). General Methods for Sampling and Gravimetric Analysis or Respirable and Inhalable Dust. http://hse.gov.uk/pubns/mdhs/pdfs/mdhs14-3.pdf.

[bib14] Helsel D.R. (2010). Summing nondetects: incorporating low-level contaminants in risk assessment. Integr. Environ. Risk Assess. Manag..

[bib15] Klasen E.M., Wills B., Naithani N., Gilman R.H., Tielsch J.M., Chiang M., Khatry S., Breysse P.N., Menya D.M., Apaka C., Carter J.E., Sherman C.B., Miranda J., Checkley W., COCINAS Trial Working Group (2015). Low correlation between household carbon monoxide and particulate matter concentrations from biomass-related pollution in three resource-poor settings. Environ. Res..

[bib16] Krieger J., Higgins D.L. (2002). Housing and health: time again for public health action. Am. J. Public Health.

[bib18] Kurmi O.P., Semple S., Steiner M., Henderson G.D., Ayres J.G. (2008). Particulate matter exposure during domestic work in Nepal. Ann. Occup. Hyg..

[bib19] Li C., Kang S., Chen P., Zhang Q., Guo J., Mi J., Basang P., Luosang Q., Smith K. (2012). Personal PM_2.5_ and indoor CO in nomadic tents using open and chimney biomass stoves on the Tibetan Plateau. Atmos. Environ..

[bib20] Lim S.S., Vos T., Flaxman A.D., Danaei G., Shibuya K., Adair-Rohani H. (2012). A comparative risk assessment of burden of disease and injury attributable to 67 risk factors and risk factor clusters in 21 regions, 1990-2010: a systematic analysis for the Global Burden of Disease Study 2010. Lancet.

[bib21] McCracken J.P., Schwarz J., Diaz A., Bruce N., Smith K.R. (2013). Longitudinal relationship between personal CO and personal PM_2.5_ among women cooking with woodfired cookstoves in Guatemala. PLoS One.

[bib22] Mustafic H., Jabre P., Caussin C., Murad M.H., Escolano S., Tafflet M., Perier M.C., Marijon E., Vernerey D., Empana J.P., Jouven X. (2012). Main air pollutants and myocardial infarction: a systematic review and meta-analysis. J. Am. Med. Assoc..

[bib23] Naeher L.P., Leaderer L.P., Smith K.R. (2000). Particulate matter and carbon monoxide in highland Guatemala: indoor and outdoor levels from traditional and improved wood stoves and gas stoves. Indoor Air.

[bib24] Naeher L.P., Smith K.R., Leaderer B.P., Neufeld L., Mage D.T. (2001). Carbon monoxide as a tracer for assessing exposures to particulate matter in wood and gas cookstove households of highland Guatemala. Environ. Sci. Technol..

[bib25] Northcross A., Chowdhury Z., McCracken J., Canuz E., Smith K.R. (2010). Estimating personal PM2.5 exposures using CO measurements in Guatemalan households cooking with wood fuel. J. Environ. Monit..

[bib26] Northcross A.L., Hwang N., Balakrishnan K., Mehta S. (2015). Assessing exposure to household air pollution in public health research and program evaluation. Ecohealth.

[bib27] Osrin D., Vaidya A., Shrestha Y., Baniya R.B., Manandhar D.S., Adhikari R.K., Filteau S., Tomkins A., Costello A.M. (2005). Effects of antenatal multiple micronutrient supplementation on birthweight and gestational duration in Nepal: double-blind, randomised controlled trial. Lancet.

[bib29] Pearce J.L., Aguialar-Villalobos M., Rathbun S.L., Naeher L.P. (2009). Residential exposures to PM_2.5_ and CO in Cusco, a high-altitude city in the Peruvian Andes: a pilot study. Archives Environ. Occup. Health.

[bib30] Pollard S.L., Williams D.L., Breysee P.N., Baron P.A., Grajeda R.H., Gilman J.J., Checkley M.W., CRONICA Cohort Study Group (2014). A cross-sectional study of determinants of indoor environmental exposures in hosueholds with and without chronic exposure to biomass fuel smoke. Environ. Health.

[bib31] Pope D.P., Mishra V., Thompson L., Siddiqui A.R., Rehfuess E.A., Weber M., Bruce N.G. (2010). Risk of low birth weight and stillbirth associated with indoor air pollution from solid fuel use in developing countries. Epidemiol. Rev..

[bib32] Rehfuess E., Mehta S., Pruss-Ustün A. (2006). Assessing household solid fuel use: multiple implications for the Millennium Development Goals. Environ. Health Perspect..

[bib33] Smith K.R. (2002). Indoor air pollution in developing countries: recommendations for research. Indoor Air.

[bib34] Smith K.R., McCracken J.P., Thompson L., Edwards R., Shields K.N., Canuz E., Bruce N. (2010). Personal child and mother carbon monoxide exposures and kitchen levels: methods and results from a randomized trial of woodfired chimney cookstoves in Guatemala (RESPIRE). J. Expo. Sci. Environ. Epidemiol..

[bib35] World Health Organization (2010). WHO Guidelines for Indoor Air Quality: Selected Pollutants. http://www.euro.who.int/__data/assets/pdf_file/0009/128169/e94535.pdf.

[bib36] World Health Organization (2014). WHO Guidelines for Indoor Air Quality: Household Fuel Combustion. http://www.who.int/indoorair/guidelines/hhfc/IAQ_HHFC_guidelines.pdf?ua=1.

[bib37] Yamamoto S.S., Louis V.R., Sié A., Sauerborn R. (2014). Biomass smoke in Burkino Faso: what is the relationship between particulate matter, carbon monoxide and kitchen characteristics?. Environ. Sci. Pollut. Res. Int..

